# Associations of Widowhood and β-Amyloid With Cognitive Decline in Cognitively Unimpaired Older Adults

**DOI:** 10.1001/jamanetworkopen.2020.0121

**Published:** 2020-02-26

**Authors:** Kelsey D. Biddle, Heidi I. L. Jacobs, Federico d’Oleire Uquillas, Benjamin S. Zide, Dylan R. Kirn, Michael R. Properzi, Dorene M. Rentz, Keith A. Johnson, Reisa A. Sperling, Nancy J. Donovan

**Affiliations:** 1 Division of Geriatric Psychiatry, Department of Psychiatry, Brigham and Women’s Hospital, Harvard Medical School, Boston, Massachusetts; 2 School for Mental Health and Neuroscience, Alzheimer Centre, Limburg, Maastricht University, Maastricht, the Netherlands; 3 Department of Radiology, Massachusetts General Hospital, Harvard Medical School, Boston; 4 Princeton Neuroscience Institute, Princeton, New Jersey; 5 Department of Neurology, Massachusetts General Hospital, Harvard Medical School, Boston; 6 Department of Neurology, Brigham and Women’s Hospital, Boston, Massachusetts; 7 Department of Psychiatry, Massachusetts General Hospital, Harvard Medical School, Boston

## Abstract

**Importance:**

To reduce the rising incidence of clinical impairment due to Alzheimer disease, it is essential to define older adults at highest risk. Widowhood may be an unrecognized factor contributing to accelerated clinical progression along the Alzheimer disease pathway among cognitively unimpaired older adults.

**Objective:**

To determine whether widowhood status and level of brain β-amyloid (ie, the Alzheimer disease pathologic protein) are additively or interactively associated with cognitive decline among cognitively unimpaired older adults.

**Design, Setting, and Participants:**

In this cohort study, 257 married, widowed, and unmarried (ie, never married, divorced, or separated) participants from the Harvard Aging Brain Study longitudinal cohort underwent baseline evaluation of neocortical β-amyloid levels using Pittsburgh compound B positron emission tomography and 4 annual cognitive assessments. Data were collected from September 2010 to February 2017 and analyzed from July 2018 to July 2019.

**Main Outcomes and Measures:**

Cognitive performance was measured using the Preclinical Alzheimer Cognitive Composite.

**Results:**

Of the 257 participants, 153 (59.5%) were women, and the mean (SD) age was 73.5 (6.1) years; 145 participants (56.4%) were married (66 [45.5%] women), 77 (30.0%) were unmarried (56 [72.7%] women), and 35 (13.6%) were widowed (31 [88.6%] women). Compared with married participants, widowed participants demonstrated worsening cognitive performance after adjusting for age, sex, socioeconomic status, depression, and β-amyloid levels (β = −0.11; 95% CI, −0.19 to −0.04; *P* = .002) with no difference observed between married and unmarried participants. Furthermore, widowed participants with higher baseline β-amyloid levels exhibited steeper cognitive decline (β = −0.22; 95% CI, −0.42 to −0.03; *P* = .02), indicating both independent and interactive associations of β-amyloid levels and widowhood with cognition. In a secondary model using dichotomous β-amyloid–marital status groupings, the rate of cognitive decline among widowed participants with high β-amyloid was nearly 3 times faster than among married participants with high β-amyloid (widowed, high β-amyloid: β, −0.33; 95% CI, −0.46 to −0.19; *P* < .001; married, high β-amyloid: β, −0.12; 95% CI, −0.18 to −0.01; *P* < .001).

**Conclusions and Relevance:**

In a sample of cognitively unimpaired older adults, being widowed was associated with accelerated β-amyloid–related cognitive decline during 3 years. Cognitively unimpaired, widowed older adults were particularly susceptible to Alzheimer disease clinical progression, emphasizing the need for increased research attention and evidenced-based interventions for this high-risk group.

## Key Points

**Question:**

Is widowhood a specific risk factor associated with more rapid cognitive decline among cognitively unimpaired older adults with higher levels of brain β-amyloid, the Alzheimer disease biomarker?

**Findings:**

In this cohort study of 257 community-dwelling cognitively unimpaired older adults, widowhood and β-amyloid were additively and interactively associated with cognitive decline. These results were independent of demographic factors, cardiovascular disease risk, depression, health-related behaviors, and social support factors.

**Meaning:**

These findings suggest that widowhood may be an understudied risk factor for cognitive decline associated with Alzheimer disease and highlight the need for increased research and clinical attention to this high-risk group.

## Teaser

This cohort study evaluates whether widowhood status and amyloid-β level are additively or interactively associated with cognitive decline among cognitively unimpaired older adults.

## Introduction

Alzheimer disease (AD) dementia is an urgent and global public health challenge, a condition that affects 50 million men and women worldwide and is projected to triple in prevalence by 2050.^1^ To reduce the incidence and consequences of AD dementia, it is essential to recognize and develop preventive treatments for older adults at high risk of AD-related cognitive decline.

Widowed older men and women are a demographic group susceptible to cognitive decline. They represent more than 11% of men and 34% of women aged 65 years or older living in the United States.^2^ Widowhood has been independently associated with declines in memory performance^3,4,5,6^ and an increased risk of incident dementia.^7^ Using prospective data from the US Health and Retirement Study, Shin et al^5^ evaluated more than 6000 married older adults and observed significantly greater cognitive decline among those who became widowed compared with those who did not, adjusting for age, race/ethnicity, sex, education, depression, other health conditions and behaviors, spouse’s medical status before death, and remarriage. Differences in cognitive performance were significant 2 years after spousal loss and worsened with longer duration of widowhood.^5^ A meta-analysis of 15 studies including 812 047 participants^7^ found that widowed men and women had a 20% greater risk of developing dementia compared with married persons during 3 to 15 years of follow-up.

Despite strong evidence linking late-life widowhood with cognitive decline and dementia, the underlying mechanisms are not well understood. Moreover, to our knowledge, the association of widowhood with cognition in individuals with biomarker evidence of early AD pathology has not been investigated.

Alzheimer disease is biologically defined by the presence of brain deposits of β-amyloid and tau pathologies, which initially accumulate while individuals have no cognitive impairment.^8,9^ Approximately 25% of cognitively unimpaired adults aged 60 years or older have elevated levels of β-amyloid, as detected by positron emission tomography (PET), and are 2 to 5 times more likely to progress to clinical impairment (ie, mild cognitive impairment or dementia) than those with low β-amyloid levels during 3 to 4 years of follow-up.^10,11^ Increased but variable rates of progression to clinical impairment among individuals with high β-amyloid levels suggest the importance of identifying other biological and clinical factors that might explain the heterogeneity of cognitive outcomes. At the same time, understanding the contribution of β-amyloid to cognitive outcomes in specific at-risk populations, such as widowed older adults, is also clinically important.

In this study, we evaluated short-term changes in cognitive performance among cognitively unimpaired, widowed older adults compared with their married counterparts and whether rates of cognitive change were further influenced by β-amyloid levels. We hypothesized an interactive association of widowhood and β-amyloid with cognition in which widowhood would be associated with worsening cognition compared with the married group, independent of age, sex, socioeconomic status, depression, and baseline β-amyloid levels, and rates of cognitive change would be accelerated among those with higher levels of β-amyloid. In secondary analyses, we tested whether the association of widowhood and cognitive change was independent of several biological, behavioral, and social support factors.

## Methods

### Participants

The sample included 257 community-dwelling cognitively unimpaired men and women participating in the Harvard Aging Brain Study, an observational, multimodal imaging study of cognition in aging and early AD. Screening procedures and inclusion and exclusion criteria for the cohort have previously been described^12^ (eAppendix in the Supplement). All participants were cognitively unimpaired at enrollment, based on a Clinical Dementia Rating^13^ global score of 0 and normal education-adjusted neuropsychological test performance.^14,15^ All participants scored below the cutoff for mild depression on the Geriatric Depression Scale (GDS), 30-item version.^16^ We included all Harvard Aging Brain Study participants who had completed 4 annual assessments in this study (eAppendix in the Supplement).

The Partners Human Research Committee approved this study. All participants provided written informed consent prior to enrollment. This report follows the Strengthening the Reporting of Observational Studies in Epidemiology (STROBE) reporting guideline.

### Marital Status

All participants reported their current marital status at the baseline study visit by selecting from the following marital categories: single, married, divorced, separated, widowed, never married, unknown, or other (eAppendix in the Supplement). For the main analyses, participants were classified into 1 of the 3 following groups: married, unmarried (ie, divorced, separated, single, or never married), or widowed. To address possible differences in cognitive outcomes within the unmarried group and to further test the specificity of the association of widowhood with cognitive change, we classified participants into the 4 following groups in supplementary models: married, divorced or separated, single or never married, and widowed.

### Cognitive Function

All participants were evaluated at 4 annual study visits using the Preclinical Alzheimer Cognitive Composite (PACC).^17^ The PACC score was computed as the mean *z* score from the Logical Memory Delayed Recall, a test of story recall, presented orally (0-25 story units)^14^; the modified Mini-Mental State Examination, a measure of global cognition (0-30 points)^15^; the Wechsler Adult Intelligence Scale–Revised Digit Symbol coding, a test of timed executive function scored during 90 seconds (0-93 symbols)^18^; and the Free and Cued Selective Reminding Test, an associative memory task measuring both free recall and recall with semantic cues, scored as the sum of the free recall and total recall components (0-96 words)^17,19^ (eAppendix in the Supplement). A higher PACC *z* score denoted better cognitive function. The PACC has been found to be sensitive to incremental declines in cognition among cognitively unimpaired older adults with elevated β-amyloid levels.^11,17^ As the Logical Memory Delayed Recall story is repeated yearly, improved PACC performance attributable to practice effects has previously been reported in Harvard Aging Brain Study participants with low β-amyloid levels.^17,20^

### Other Clinical Measures

Socioeconomic status was assessed using the Two-Factor Hollingshead score, on which a higher score indicates lower socioeconomic status.^21^ Depression was measured at baseline and annually using the 30-item GDS. History of any episode of depressive disorder was scored as absent or present. Cardiovascular disease risk score was derived from a weighted sum of age, sex, antihypertensive treatment, systolic blood pressure, body mass index (calculated as weight in kilograms divided by height in meters squared), history of diabetes, and current cigarette smoking status.^22,23^

Level of social engagement was assessed at baseline using 5 questions from the Community Healthy Activities Model Program for Seniors questionnaire, which measures time spent per week with family, friends, and in community activities (range, 0-30).^12,24^ Analogous scoring for 27 items on the Community Healthy Activities Model Program for Seniors pertaining to sports, exercise, and dance were combined to calculate a baseline physical activity score (range, 0-162).

Level of emotional support from children, relatives, and friends was assessed by 3 questionnaire items,^25^ which asked participants to specify numbers of close relationships with children, relatives, and friends during the fourth annual assessment (eAppendix in the Supplement). As these data were not collected at earlier assessments, these responses were used as proxy baseline data for the purpose of this study.

### Hippocampal Volume

All magnetic resonance imaging was performed at the Massachusetts General Hospital, Athinoula A. Martinos Center for Biomedical Imaging on a 3-T imaging system (TIM Trio; Siemens) with a 12-channel phased-array head coil. The protocol consisted of a T1-weighted volumetric magnetization–prepared rapid-acquisition gradient-echo sequence (repetition time, 2300 milliseconds; echo time, 2.95 milliseconds; inversion time, 900 milliseconds; flip angle, 9°; 1.05 × 1.05 × 1.2 mm resolution). We processed and quality-assessed T1-weighted images using FreeSurfer version 6.0.0 with the software package’s default automated reconstruction protocol, as described previously.^26,27,28^ Bilateral hippocampal volumes were extracted and adjusted for the estimated intracranial volume (eTIV) using the following equation^29^: adjusted hippocampal volume = raw hippocampal volume − *b*(eTIV − mean eTIV), in which *b* indicates the regression coefficient when hippocampal volume is regressed against eTIV.

### Pittsburgh Compound B PET

We measured β-amyloid burden using Pittsburgh compound B (PiB)-PET protocols, as previously described.^30^ Briefly, PET data were reconstructed and attenuation-corrected using standard Siemens software. We calculated PiB retention^31^ using a gray matter cerebellum reference region and applied a geometric transfer matrix partial volume correction.^32^ Neocortical β-amyloid deposition was quantified using an aggregate partial volume correction distribution volume ratio from a set of FreeSurfer regions including frontal, lateral parietal, lateral temporal, and retrosplenial cortices.^30^ Dichotomous high β-amyloid and low β-amyloid groups were defined by Gaussian mixture modeling, resulting in a threshold value of greater than 1.32 PiB distribution volume ratio for the high β-amyloid group.^33^ A continuous measure of β-amyloid was used in the main analyses.

### Statistical Analysis

All statistical analyses were performed using R software version 3.6.1 (R Project for Statistical Computing). Baseline values for demographic, clinical, and imaging variables were compared across marital groups using analysis of variance, Kruskal-Wallis, Tukey, χ^2^, and Fisher exact tests.

In the first main linear mixed-effects model, we examined the association of marital status with longitudinal PACC scores, including marital status, time, and its interaction as fixed effects and individual intercepts and slopes for time in years as random effects. The unmarried and widowed groups were compared with the married reference group to estimate differences in longitudinal PACC scores using the maximum likelihood estimation, controlling for age, sex, socioeconomic status, depression history, depressive symptoms, β-amyloid level, and their interactions with time. A second main model examined the association of the 3-way multiplicative interaction of marital status, continuous β-amyloid level, and time with longitudinal PACC scores, controlling for the same covariate-time interactions and main terms.

Interactions of β-amyloid levels and marital status were further evaluated in a secondary model using 6 marital status and dichotomous β-amyloid groups to estimate longitudinal PACC scores, controlling for the same covariates. The 6 groups were married, low β-amyloid (99 participants; reference group); married, high β-amyloid (44 participants); unmarried, low β-amyloid (53 participants); unmarried, high β-amyloid (23 participants); widowed, low β-amyloid (28 participants); and widowed, high β-amyloid (7 participants).

Secondary analyses probed the specificity of the association of marital status with cognitive and neurobehavioral outcomes. These analyses, analogous to the main models, estimated longitudinal scores for each of the PACC component tests (rather than the composite PACC score) or for longitudinal GDS scores as separate dependent variables, with marital status and its interaction with time or its interaction with time and β-amyloid level as the variables of interest. Additional secondary models adjusted for biological factors (ie, hippocampal volume and cardiovascular risk score), behavioral factors (ie, level of physical exercise and social engagement), or social support (ie, numbers of close relationships) to test whether these covariates contributed to the association of widowhood with cognition.

For linear mixed-effects models, we report unstandardized coefficient estimates (β) with 95% CIs, *t* statistics or effect sizes (ie, Cohen *d*), and *P* values. We considered *P* < .05 statistically significant, and all tests were 2-tailed. Residual-vs-fits and normality plots were evaluated for each variable and model to ensure that distributions reasonably satisfied model assumptions of normality and homoscedasticity.

## Results

### Associations of Baseline Variables

Of 257 participants, 153 (59.5%) were women, and the mean (SD) age was 73.5 (6.1) years; 145 participants (56.4%) were married (66 [45.5%] women), 77 (30.0%) were unmarried (56 [72.7%] women), and 35 (13.6%) were widowed (31 [88.6%] women). Baseline data and statistical comparisons across marital status groups are shown in Table 1. Compared with the married group, the widowed group was older (mean [SD] age, 73.3 [5.8] years vs 77.6 [6.6] years; *P* < .001), had proportionally more women (66 [45.5%] vs 56 [72.7%]; *P* < .001), and had lower socioeconomic status (mean [SD] Hollingshead score, 24.4 [14.0] vs 31.4 [13.7]; *P* = .03). The widowed group was also older than the unmarried group (mean [SD] age, 77.6 [6.6] years vs 72.1 [5.5]; *P* < .001) but did not differ from the unmarried group by sex or socioeconomic status. There were no baseline group differences in mean PiB, GDS score, PACC *z* scores, or depression history. Compared with the married group, the widowed group had higher social engagement scores (mean [SD], 7.5 [4.0] vs 9.5 [3.9]; *P* = .04), but the groups did not differ in physical activity or close relationship scores (eTable 1 in the Supplement). There were no significant group differences in lifetime prevalence of hypertension, diabetes, cardiovascular or cerebrovascular conditions, hearing loss, alcohol or substance use disorders, or current smoking or alcohol use (eTable 2 in the Supplement).

**Table 1.** Baseline Demographic, Clinical, and Imaging Data

Characteristic	Mean (SD)				*P* Value			
	Overall (N = 257)	Married (n = 145)	Unmarried (n = 77)	Widowed (n = 35)	UM-M	W-M	W-UM	
Age, y	73.5 (6.1)	73.3 (5.8)	72.1 (5.5)	77.6 (6.6)	.36	<.001	<.001	
Women, No. (%)	153 (59.5)	66 (45.5)	56 (72.7)	31 (88.6)	<.001	<.001		.10
Hollingshead score^a^	26.5 (14.5)	24.4 (14.0)	28.1 (15.1)	31.4 (13.7)	.16	.03	.50	
Geriatric Depression Scale score	3.1 (2.8)	2.8 (2.5)	3.6 (3.1)	3.4 (3.4)	.11	.48	.94	
Depression history, No. (%)	39 (15.2)	20 (13.8)	13 (16.9)	6 (17.1)	.68	.82		>.99
β-amyloid burden, neocortical PiB PVC DVR^b^	1.35 (0.38)	1.36 (0.40	1.35 (0.37)	1.30 (0.33)	.99	.63	.74	
PACC *z* score	0.03 (0.63)	0.08 (0.60)	0.02 (0.66)	−0.13 (0.65)	.79	.18	.47	
MMSE score	29.1 (1.0)	29.1 (0.9)	29.1 (1.1)	28.8 (1.3)	.92	.17	.35	
FCSRT score^c^	47.7 (0.9)	47.7 (0.8)	47.9 (1.1)	47.6 (1.2)	>.99	.79	.84	
Logical Memory score	13.9 (3.2)	13.7 (2.8)	14.2 (3.5)	14.6 (3.6)	.58	.28	.77	
Digit Symbol Substitution Test score^d^	48.3 (9.9)	50.0 (9.1)	46.7 (11.4)	44.1 (8.8)	.09	<.001	.45	
Follow-up time, y	3.1 (0.2)	3.1 (0.2)	3.1 (0.2)	3.1 (0.2)	.40	.94	.82	

Abbreviations: DVR, distribution volume ratio; FCSRT, Free and Cued Selective Reminding Test; MMSE, Mini-Mental State Examination; PACC, Preclinical Alzheimer Cognitive Composite; PiB, Pittsburgh compound B; PVC, partial volume correction; UM-M, unmarried compared with married; W-M, widowed compared with married; W-UM, widowed compared with unmarried.

^a^ Higher score indicates lower socioeconomic status.

^b^ Available for 254 participants.

^c^ Available for 235 participants.

^d^ Available for 256 participants.

### Longitudinal Cognitive Function

**Figure 1.**
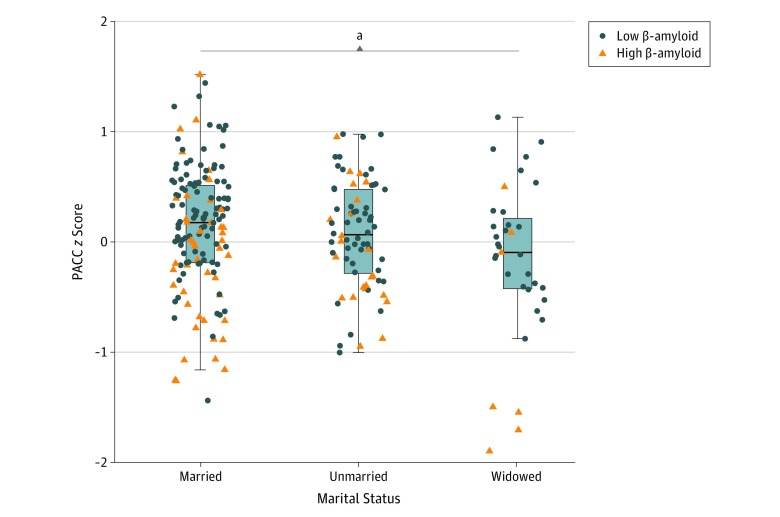
Three-Year Change in Preclinical Alzheimer Cognitive Composite (PACC) *z* Score by Marital Status Group Change in PACC scores was calculated for each participant as the difference between PACC *z* scores at baseline and 3 years later. Blue circles represent participants who had low β-amyloid levels at baseline, and orange triangles represent those with high β-amyloid levels at baseline, based on the standard cutoff of 1.32 Pittsburgh compound B distribution volume ratio. ^a^Married group compared with widowed group, *P* = .03.

During the 3 years of observation, mean (SD) change in unadjusted PACC *z* score was 0.13 (0.56) for the married group, 0.07 (0.48) for the unmarried group and −0.16 (0.72) for the widowed group (unmarried compared with married: *t*_177_ = 0.90; *P* = .37; Cohen *d* = 0.12; widowed compared with married: *t*_44_ = 2.25; *P* = .03; Cohen *d* = 0.50; widowed compared with unmarried: *t*_48_ = −1.71; *P* = .09; Cohen *d* = 0.40) (Figure 1).

**Figure 2.**
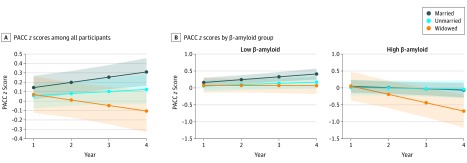
Association of Preclinical Alzheimer Cognitive Composite (PACC) *z *Score With Marital Status A, PACC scores declined in widowed but not unmarried participants during 3 years compared with married participants, controlling for age, sex, socioeconomic status, depression history, depressive symptoms, neocortical β-amyloid level, and their associations by time. B, Predicted trajectories for marital status groups are shown separately for low–β-amyloid and high–β-amyloid groups, controlling for the same covariates. β-amyloid groups are based on the standard cutoff of 1.32 Pittsburgh compound B distribution volume ratio. Shaded areas represent 95% CIs.

In the first mixed-effects model, cognitive performance on PACC declined in the widowed group, differing significantly from the married group (β, −0.11; 95% CI, −0.19 to −0.04; *P* = .002) (Table 2 and Figure 2A). Longitudinal PACC scores did not differ between the married and unmarried groups (β, −0.03; 95% CI, −0.08 to 0.02; *P* = .21) (Table 2 and Figure 2A). Higher β-amyloid level (β, −0.14; 95% CI, −0.19 to −0.08; *P* < .001) and older age (β, −0.004; 95% CI, −0.01 to −0.00004; *P* = .045) were also associated with declining PACC scores in this model (Table 2).

**Table 2.** Linear Mixed-Effects Model for Association of Longitudinal Cognition With Marital Status Groups^a^

Model	β Estimate (95% CI)	*t* Value	*P* Value
**Model 1**			
Widowed × time^b^	−0.11 (−0.19 to −0.04)	−3.06	.002
Unmarried × time^b^	−0.03 (−0.08 to 0.02)	−1.26	.21
Baseline β-amyloid level × time	−0.14 (−0.19 to −0.08)	−4.53	<.001
Baseline age × time	−0.004 (−0.01 to −0.00004)	−1.97	.045
**Model 2**			
Widowed and β-amyloid level × time^b^	−0.22 (−0.42 to −0.03)	−2.25	.02
Unmarried and β-amyloid level × time^b^	−0.01 (−0.14 to 0.12)	−0.12	.91
Baseline β-amyloid level × time	−0.11 (−0.19 to −0.04)	−2.94	.003
Baseline age × time	−0.004 (−0.01 to 0.0002)	−1.82	.07

^a^ Models included 254 participants and 1015 observations and were adjusted for age, sex, socioeconomic status, depression history, depressive symptoms, and their interactions with time. Results for factors of interest and covariates associated with longitudinal cognition (*P* < .10) are shown.

^b^ Reference group was married group.

In the second main model, β-amyloid–associated PACC decline was steeper in the widowed group compared with the married group (β, −0.22; 95% CI, −0.42 to −0.03; *P* = .02) but did not differ between the unmarried and married groups (β, −0.01; 95% CI, −0.14 to 0.12; *P* = .91) (Table 2). Results indicated a specific and synergistic association of the widowed group and higher β-amyloid level with PACC decline beyond their independent associations.

Results were consistent in models using β-amyloid as a dichotomous grouping variable. Longitudinal PACC scores worsened in the widowed, low–β-amyloid group compared with the married, low–β-amyloid reference group (β, −0.09; 95% CI, −0.17 to −0.009; *P* = .03) but did not differ between the unmarried, low–β-amyloid and married, low–β-amyloid groups (β, −0.05; 95% CI, −0.11 to 0.01; *P* = .12) (Figure 2B; eTable 3 in the Supplement). Moreover, longitudinal PACC scores were worse in all 3 high–β-amyloid groups compared with the married, low–β-amyloid reference group (married, high β-amyloid: β, −0.12; 95% CI, −0.18 to −0.06; *P* < .001; unmarried, high β-amyloid: β, −0.10; 95% CI, −0.18 to −0.02; *P* = .01; widowed, high β-amyloid: β, −0.33; 95% CI, −0.46 to −0.19; *P* < .001) (Figure 2B; eTable 3 in the Supplement). These β estimates indicate that the rate of PACC decline in the widowed, high–β-amyloid group was nearly 3-fold faster than in the married, high β-amyloid group.

Results for the main models remained significant in sensitivity analyses removing the widowed participant with lowest cognitive function and trajectory or removing all participants who changed marital status (data not shown). Models using 4 rather than 3 marital categories also showed consistent results for the widowed and unmarried subgroups (eTable 4, eTable 5, and eFigure in the Supplement).

### Depression and Cognition Secondary Analyses

Widowhood, but not depression, was associated with declining cognition in the main analyses (GDS × time: β, 0.004; 95% CI, −0.004 to 0.01; *P* = .29; depression history × time: β, 0.02; 95% CI −0.05 to 0.08; *P* = .65). In a secondary analysis examining the association of marital status with depression as the outcome, no association was found between marital status and depression (ie, GDS) scores over time (eTable 6 in the Supplement).

Examining marital status with each PACC component test as separate outcomes, widowhood was significantly associated with worsening performance on the Logical Memory Test (β, −0.53; 95% CI, −0.96 to −0.09; *P* = .02) and the Free and Cued Selective Reminding Test (β, −0.27; 95% CI, −0.48 to −0.07; *P* = .01). Associations did not reach significance with other tests (eTable 7 in the Supplement).

### Adjusting for Biological Factors

The association of widowhood with declining PACC scores remained significant in models that included β-amyloid level as well as cardiovascular disease risk score, hippocampal volume, and their interactions with time. Results of this model indicated independent associations of widowhood (β, −0.12; 95% CI, −0.19 to −0.04; *P* = .005), higher cardiovascular disease risk score (β, −0.003; 95% CI, −0.005 to −0.0005; *P* = .02), and greater β-amyloid level (β, −0.13; 95% CI, −0.19 to −0.07; *P* < .001) but not hippocampal volume (β, <0.001; 95% CI, −0.00003 to 0.00004; *P* = .94) with worsening PACC scores (Table 3).

**Table 3.** Linear Mixed-Effects Model for Association of Longitudinal Cognition With Marital Status Groups Adjusting for Biological, Behavioral, or Social Support Factors^a^

Model	β Estimate (95% CI)	*t* Value	*P* Value
**Model 3a**			
Widowed × time^b^	−0.12 (−0.19 to −0.04)	−2.81	.005
Baseline β-amyloid level × time	−0.13 (−0.19 to −0.07)	−3.9	<.001
Cardiovascular disease × time	−0.003 (−0.005 to −0.0005)	−2.35	.02
Baseline hippocampal volume × time	<0.001 (−0.00003 to 0.00004)	0.07	.94
**Model 3b**			
Widowed × time^b^	−0.13 (−0.19 to −0.06)	−3.57	<.001
Baseline β-amyloid level × time	−0.14 (−0.19 to −0.08)	−4.76	<.001
Social engagement × time	−0.0003 (−0.005 to 0.005)	−0.13	.90
Physical activity × time	0.001 (−0.0004 to 0.003)	1.46	.14
**Model 3c**			
Widowed × time^b^	−0.10 (−0.17 to −0.04)	−3.04	.002
Baseline β-amyloid level × time	−0.14 (−0.19 to −0.08)	−4.92	<.001
Close relationships × time	0.004 (−0.0002 to 0.008)	1.84	.07

^a^ Models adjusted for age, sex, socioeconomic status, depression history, depressive symptoms, and their interactions with time. Model 3a included 162 participants and 648 observations; 3b, 191 participants and 763 observations; and 3c, 208 participants and 832 observations. Results for factors of interest are shown.

^b^ Reference group was married group.

### Adjusting for Behavioral or Social Support Factors

The association of widowhood with PACC scores remained significant when covarying for physical activity scores, social engagement scores, and their interaction with time (β, −0.13; 95% CI, −0.19 to −0.06; *P* < .001), and these additional terms were not associated with longitudinal PACC scores (physical activity: β, 0.001; 95% CI, −0.0004 to 0.003; *P* = .14; social engagement: β, −0.0003; 95% CI, −0.005 to 0.005; *P* = .90) (Table 3). The widowhood × time association with PACC scores also remained significant in a model covarying for number of close relationships and its interaction with time (β, −0.10; 95% CI, −0.17 to −0.04; *P* = .002). The close relationships × time interaction was not associated with PACC scores (β, 0.004; 95% CI, −0.0002 to 0.008; *P* = .07) (Table 3).

## Discussion

The purpose of this study was to evaluate the associations of widowhood, β-amyloid levels, and cognitive function in a sample of cognitively unimpaired older adults. High β-amyloid levels were associated with cognitive decline in all marital groups during 3 years of observation, with a much steeper rate of decline among participants who were widowed. The widowed, high–β-amyloid group declined 3 times faster than the married, high–β-amyloid group, independent of age, sex, socioeconomic status, and depression. Cognitive trajectories also differed between married and widowed participants with low β-amyloid, pointing to both independent and synergistic associations of widowhood and β-amyloid levels with cognition over time. There were no differences in cognitive outcomes between married and unmarried (ie, nonwidowed) participants. These findings illustrate the importance of widowhood as a clinically relevant risk factor for cognitive decline, and they provide new evidence that widowed men and women are a distinct subgroup of older adults who are particularly susceptible to progression in early AD.

Both risk of widowhood and risk of AD dementia increase with advancing age, particularly among women. Recent US census data have documented that widows include 6.4% of men and 19.5% of women aged 65 to 74 years, 14.7% of men and 42.9% of women aged 75 to 84 years, and 35.3% of men and 71.9% of women aged 85 years or older.^2^ Thus, widowhood may contribute to the excess AD dementia risk observed in older women, but this association is largely unrecognized. Importantly, these data support that both male and female widowed older adults are a large and readily identified demographic group who may be at high risk of AD-related cognitive decline and merit both research and clinical attention.

Death of a spouse is considered to be among the most stressful life events.^34^ Early sequelae include painful feelings of loss,^35^ sadness,^36^ and sleep disturbance, particularly in the context of greater depressive symptoms.^37,38^ Physiologic arousal, manifested as higher heart rate,^39^ higher systolic blood pressure,^39^ and elevation in morning blood cortisol level,^40^ can also occur and persist for months after spousal loss.^41^ Other reported changes include increased platelet activation,^42^ higher levels of proinflammatory cytokines,^43^ and alterations in cellular immune response.^40,44^ It is well established that risk of myocardial infarction, stroke, nonacute coronary syndrome, and pulmonary embolism are elevated in the days and months following spousal loss.^45^ These early behavioral, cardiovascular, and inflammatory alterations may plausibly interact or act in parallel with neurodegenerative processes, such as AD, to lower the threshold for cognitive decline; however, relatively little is known of behavioral or physiologic sequelae beyond this early period and how such changes might specifically relate to ongoing cognitive decline.^46^

We found no evidence that the association of widowhood with cognitive decline was mediated by depression history or depressive symptom burden in this sample. This is consistent with other observational studies, which have reported independent associations of widowhood and depression with cognitive decline^3,6^ and risk of cognitive impairment.^47^

In this select, relatively healthy sample, widowed participants did not differ from other marital groups by health conditions or behaviors, age-adjusted hippocampal volumes, level of β-amyloid, or cardiovascular disease risk scores at baseline. Despite these baseline similarities, we found evidence that widowhood, level of β-amyloid, and cardiovascular disease risk score had independent and cumulative associations with longitudinal cognition at the individual level. Widowhood had a unique association with cognitive decline that was not accounted for by these other known biological risk factors.

Death of a spouse can pose a critical loss of intimacy, meaningfulness, companionship, and everyday support. Close relationships have the potential to buffer against the physiological and psychological effects of stress and provide opportunities for cognitive stimulation.^25^ Close family relationships (eg, having a child or living sibling) have previously been found to affect cognitive outcomes in widowed people.^5,48^

We found no association for number of close relationships, social engagement, or physical activity with longitudinal cognition. Negative findings in these secondary analyses should be interpreted cautiously because of the limits of sample size, duration of follow-up, and the overall cognitive stability of the sample.

### Limitations

This study has limitations. Widowed participants in the study were healthier than in the general population, which may have led to an underestimation of the association of widowhood with cognitive decline. We were unable to investigate sex-related differences within the widowed group, given that nearly all participants were women. These analyses did not take into account nonmarried partners, information on earlier marriages, marriage quality, cause of spousal death, or caregiver status. Furthermore, our study lacked inflammatory biomarker, sleep, or other physiologic data to elucidate possible mediators or moderators of the association of widowhood with cognitive decline. Lastly, as β-amyloid neuroimaging is primarily used for research purposes at this time, our findings do not have immediate relevance to the clinical care of older individuals. However, these findings may inform the design of new detection and prevention initiatives for unimpaired older adults using scalable, biomarker-based diagnostic tests for AD.

## Conclusions

In this cohort study, we examined a cognitively unimpaired sample and observed declining cognitive function among widowed older adults compared with their married peers during 3 years, with more rapid declines in participants with higher levels of brain β-amyloid. Cardiovascular disease risk profile was further associated with cognitive trajectories among widowed participants. Widowhood is an underrecognized risk factor associated with AD-related cognitive decline and impairment. Further research and intervention strategies are needed to ameliorate psychosocial and biological processes underlying cognitive decline in widowed older adults.

## Notes

Supplement. **eAppendix. **Supplemental Descriptions of Participants, Marital Status, and Emotional Support Score **eTable 1. **Social Engagement, Physical Activity, and Close Relationship Data for the Sample **eTable 2. **Baseline Medical, Psychiatric, and Health Behavior Data for the Sample **eTable 3. **Linear Mixed-Effects Model for Association of Longitudinal Cognition With Dichotomous PiB-Marital Status Groups **eTable 4. **Linear Mixed-Effects Model for Association of Longitudinal Cognition With 4 Marital Status Groups **eTable 5. **Linear Mixed-Effects Model for Association of Longitudinal Cognition With 4 Marital Status Groups Interacting With PiB **eTable 6. **Linear Mixed-Effects Models for the Association of Longitudinal Geriatric Depression Scores With 3 Marital Status Groups **eTable 7. **Linear Mixed-Effects Models for the Association of PACC Component Test Performance With 3 Marital Status Groups **eFigure. **Association of Longitudinal Cognition With 4 Marital Status Groups **eReferences.**

## References

[1] Patterson C World Alzheimer Report 2018: the state of the art of dementia research: new frontiers. https://www.alz.co.uk/research/WorldAlzheimerReport2018.pdf. Accessed January 17, 2020.

[2] Roberts AW, Ogunwole SU, Blakeslee L, Rabe MA The population 65 years and older in the United States: 2016. https://www.census.gov/content/dam/Census/library/publications/2018/acs/ACS-38.pdf. Accessed January 17, 2020.

[3] Aartsen MJ, van Tilburg T, Smits CH, Knipscheer KCPM A longitudinal study of the impact of physical and cognitive decline on the personal network in old age. J Soc Pers Relat. 2004;21(2):-. doi:10.1177/0265407504041386

[4] Karlamangla AS, Miller-Martinez D, Aneshensel CS, Seeman TE, Wight RG, Chodosh J Trajectories of cognitive function in late life in the United States: demographic and socioeconomic predictors. Am J Epidemiol. 2009;170(3):331-342. doi:10.1093/aje/kwp154 19605514 PMC2727175

[5] Shin SH, Kim G, Park S Widowhood status as a risk factor for cognitive decline among older adults. Am J Geriatr Psychiatry. 2018;26(7):778-787. doi:10.1016/j.jagp.2018.03.013 29748078

[6] Zhang Z, Li LW, Xu H, Liu J Does widowhood affect cognitive function among Chinese older adults? SSM Popul Health. 2018;7:100329. doi:10.1016/j.ssmph.2018.100329 30581964 PMC6293047

[7] Sommerlad A, Ruegger J, Singh-Manoux A, Lewis G, Livingston G Marriage and risk of dementia: systematic review and meta-analysis of observational studies. J Neurol Neurosurg Psychiatry. 2018;89(3):231-238. doi:10.1136/jnnp-2017-316274 29183957 PMC5869449

[8] Sperling RA, Aisen PS, Beckett LA, Toward defining the preclinical stages of Alzheimer’s disease: recommendations from the National Institute on Aging–Alzheimer’s Association workgroups on diagnostic guidelines for Alzheimer’s disease. Alzheimers Dement. 2011;7(3):280-292. doi:10.1016/j.jalz.2011.03.003 21514248 PMC3220946

[9] Jack CR Jr, Bennett DA, Blennow K, ; Contributors NIA-AA Research Framework: toward a biological definition of Alzheimer’s disease. Alzheimers Dement. 2018;14(4):535-562. doi:10.1016/j.jalz.2018.02.018 29653606 PMC5958625

[10] Burnham SC, Bourgeat P, Doré V, ; AIBL Research Group Clinical and cognitive trajectories in cognitively healthy elderly individuals with suspected non-Alzheimer’s disease pathophysiology (SNAP) or Alzheimer’s disease pathology: a longitudinal study. Lancet Neurol. 2016;15(10):1044-1053. doi:10.1016/S1474-4422(16)30125-9 27450471

[11] Donohue MC, Sperling RA, Petersen R, Sun CK, Weiner MW, Aisen PS; Alzheimer’s Disease Neuroimaging Initiative Association between elevated brain amyloid and subsequent cognitive decline among cognitively normal persons. JAMA. 2017;317(22):2305-2316. doi:10.1001/jama.2017.6669 28609533 PMC5736301

[12] Biddle KD, d’Oleire Uquillas F, Jacobs HIL, Social engagement and amyloid-β-related cognitive decline in cognitively normal older adults. Am J Geriatr Psychiatry. 2019;27(11):1247-1256. doi:10.1016/j.jagp.2019.05.005 31248770 PMC6778491

[13] Morris JC The Clinical Dementia Rating (CDR): current version and scoring rules. Neurology. 1993;43(11):2412-2414. doi:10.1212/WNL.43.11.2412-a 8232972

[14] Abikoff H, Alvir J, Hong G, Logical memory subtest of the Wechsler Memory Scale: age and education norms and alternate-form reliability of two scoring systems. J Clin Exp Neuropsychol. 1987;9(4):435-448. doi:10.1080/01688638708405063 3597734

[15] Folstein MF, Folstein SE, McHugh PR “Mini-Mental State”: a practical method for grading the cognitive state of patients for the clinician. J Psychiatr Res. 1975;12(3):189-198. doi:10.1016/0022-3956(75)90026-6 1202204

[16] Yesavage JA, Brink TL, Rose TL, Development and validation of a Geriatric Depression Screening scale: a preliminary report. J Psychiatr Res. 1982-1983;17(1):37-49. doi:10.1016/0022-3956(82)90033-4 7183759

[17] Mormino EC, Papp KV, Rentz DM, Early and late change on the preclinical Alzheimer’s cognitive composite in clinically normal older individuals with elevated amyloid β. Alzheimers Dement. 2017;13(9):1004-1012. doi:10.1016/j.jalz.2017.01.018 28253478 PMC5573651

[18] Weschler D WMS-R Weschler Memory Scale Revised Manual. New York, NY: Harcourt Brace Jovanovich, Inc; 1987.

[19] Grober E, Hall C, Sanders AE, Lipton RB Free and cued selective reminding distinguishes Alzheimer’s disease from vascular dementia. J Am Geriatr Soc. 2008;56(5):944-946. doi:10.1111/j.1532-5415.2008.01652.x 18454754 PMC2735231

[20] Buckley RF, Schultz AP, Hedden T, Functional network integrity presages cognitive decline in preclinical Alzheimer disease. Neurology. 2017;89(1):29-37. doi:10.1212/WNL.0000000000004059 28592457 PMC5496516

[21] Hollingshead AB Two Factor Index of Social Position. New Haven, CT: Yale University Press; 1957.

[22] D’Agostino RB Sr, Vasan RS, Pencina MJ, General cardiovascular risk profile for use in primary care: the Framingham Heart Study. Circulation. 2008;117(6):743-753. doi:10.1161/CIRCULATIONAHA.107.699579 18212285

[23] Rabin JS, Schultz AP, Hedden T, Interactive associations of vascular risk and β-amyloid burden with cognitive decline in clinically normal elderly individuals: findings from the Harvard Aging Brain Study. JAMA Neurol. 2018;75(9):1124-1131. doi:10.1001/jamaneurol.2018.1123 29799986 PMC6143121

[24] Stewart AL, Mills KM, King AC, Haskell WL, Gillis D, Ritter PL CHAMPS physical activity questionnaire for older adults: outcomes for interventions. Med Sci Sports Exerc. 2001;33(7):1126-1141. doi:10.1097/00005768-200107000-00010 11445760

[25] Seeman TE, Lusignolo TM, Albert M, Berkman L Social relationships, social support, and patterns of cognitive aging in healthy, high-functioning older adults: MacArthur studies of successful aging. Health Psychol. 2001;20(4):243-255. doi:10.1037/0278-6133.20.4.243 11515736

[26] Dale AM, Fischl B, Sereno MI Cortical surface-based analysis: segmentation and surface reconstruction. Neuroimage. 1999;9(2):179-194. doi:10.1006/nimg.1998.0395 9931268

[27] Fischl B, Sereno MI, Dale AM Cortical surface-based analysis: inflation, flattening, and a surface-based coordinate system. Neuroimage. 1999;9(2):195-207. doi:10.1006/nimg.1998.0396 9931269

[28] Jacobs HIL, Hedden T, Schultz AP, Structural tract alterations predict downstream tau accumulation in amyloid-positive older individuals. Nat Neurosci. 2018;21(3):424-431. doi:10.1038/s41593-018-0070-z 29403032 PMC5857215

[29] Mormino EC, Betensky RA, Hedden T, Synergistic effect of β-amyloid and neurodegeneration on cognitive decline in clinically normal individuals. JAMA Neurol. 2014;71(11):1379-1385. doi:10.1001/jamaneurol.2014.2031 25222039 PMC4293023

[30] Johnson KA, Gregas M, Becker JA, Imaging of amyloid burden and distribution in cerebral amyloid angiopathy. Ann Neurol. 2007;62(3):229-234. doi:10.1002/ana.21164 17683091

[31] Price JC, Klunk WE, Lopresti BJ, Kinetic modeling of amyloid binding in humans using PET imaging and Pittsburgh Compound-B. J Cereb Blood Flow Metab. 2005;25(11):1528-1547. doi:10.1038/sj.jcbfm.9600146 15944649

[32] Greve DN, Svarer C, Fisher PM, Cortical surface-based analysis reduces bias and variance in kinetic modeling of brain PET data. Neuroimage. 2014;92:225-236. doi:10.1016/j.neuroimage.2013.12.021 24361666 PMC4008670

[33] Hanseeuw BJ, Betensky RA, Schultz AP, Fluorodeoxyglucose metabolism associated with tau-amyloid interaction predicts memory decline. Ann Neurol. 2017;81(4):583-596. doi:10.1002/ana.24910 28253546 PMC5404378

[34] Holmes TH, Rahe RH The Social Readjustment Rating Scale. J Psychosom Res. 1967;11(2):213-218. doi:10.1016/0022-3999(67)90010-4 6059863

[35] Fried EI, Bockting C, Arjadi R, From loss to loneliness: the relationship between bereavement and depressive symptoms. J Abnorm Psychol. 2015;124(2):256-265. doi:10.1037/abn0000028 25730514

[36] Wilcox S, Evenson KR, Aragaki A, Wassertheil-Smoller S, Mouton CP, Loevinger BL The effects of widowhood on physical and mental health, health behaviors, and health outcomes: the Women’s Health Initiative. Health Psychol. 2003;22(5):513-522. doi:10.1037/0278-6133.22.5.513 14570535

[37] Reynolds CF III, Hoch CC, Buysse DJ, Electroencephalographic sleep in spousal bereavement and bereavement-related depression of late life. Biol Psychiatry. 1992;31(1):69-82. doi:10.1016/0006-3223(92)90007-M 1543799

[38] Pasternak RE, Reynolds CF III, Hoch CC, Sleep in spousally bereaved elders with subsyndromal depressive symptoms. Psychiatry Res. 1992;43(1):43-53. doi:10.1016/0165-1781(92)90140-X 1438616

[39] Buckley T, Mihailidou AS, Bartrop R, Haemodynamic changes during early bereavement: potential contribution to increased cardiovascular risk. Heart Lung Circ. 2011;20(2):91-98. doi:10.1016/j.hlc.2010.10.073 21147029

[40] Irwin M, Daniels M, Risch SC, Bloom E, Weiner H Plasma cortisol and natural killer cell activity during bereavement. Biol Psychiatry. 1988;24(2):173-178. doi:10.1016/0006-3223(88)90272-7 3390497

[41] Buckley T, Sunari D, Marshall A, Bartrop R, McKinley S, Tofler G Physiological correlates of bereavement and the impact of bereavement interventions. Dialogues Clin Neurosci. 2012;14(2):129-139. 22754285 10.31887/DCNS.2012.14.2/tbuckley PMC3384441

[42] Buckley T, Morel-Kopp MC, Ward C, Inflammatory and thrombotic changes in early bereavement: a prospective evaluation. Eur J Prev Cardiol. 2012;19(5):1145-1152. doi:10.1177/1741826711421686 21900365

[43] Fagundes CP, Brown RL, Chen MA, Grief, depressive symptoms, and inflammation in the spousally bereaved. Psychoneuroendocrinology. 2019;100:190-197. doi:10.1016/j.psyneuen.2018.10.006 30368120 PMC6889080

[44] Gerra G, Monti D, Panerai AE, Long-term immune-endocrine effects of bereavement: relationships with anxiety levels and mood. Psychiatry Res. 2003;121(2):145-158. doi:10.1016/S0165-1781(03)00255-5 14656449

[45] Carey IM, Shah SM, DeWilde S, Harris T, Victor CR, Cook DG Increased risk of acute cardiovascular events after partner bereavement: a matched cohort study. JAMA Intern Med. 2014;174(4):598-605. doi:10.1001/jamainternmed.2013.14558 24566983

[46] Stroebe M, Schut H, Stroebe W Health outcomes of bereavement. Lancet. 2007;370(9603):1960-1973. doi:10.1016/S0140-6736(07)61816-9 18068517

[47] Bae JB, Kim YJ, Han JW, Incidence of and risk factors for Alzheimer’s disease and mild cognitive impairment in Korean elderly. Dement Geriatr Cogn Disord. 2015;39(1-2):105-115. doi:10.1159/000366555 25401488

[48] Sundström A, Westerlund O, Mousavi-Nasab H, Adolfsson R, Nilsson LG The relationship between marital and parental status and the risk of dementia. Int Psychogeriatr. 2014;26(5):749-757. doi:10.1017/S1041610213002652 24451183

