# Supplementary material for: Associations of Widowhood and β-Amyloid With Cognitive Decline in Cognitively Unimpaired Older Adults
Source: JAMA Netw Open. 2020 Feb 26;3(2):e200121. doi: 10.1001/jamanetworkopen.2020.0121 (PMC7099624; doi:10.1001/jamanetworkopen.2020.0121)
Supplement: Supplement. — eAppendix. Supplemental Descriptions of Participants, Marital Status, and Emotional Support Score eTable 1. Social Engagement, Physical Activity, and Close Relationship Data for the Sample eTable 2. Baseline Medical, Psychiatric, and Health Behavior Data for the Sample eTable 3. Linear Mixed-Effects Model for Association of Longitudinal Cognition With Dichotomous PiB-Marital Status Groups eTable 4. Linear Mixed-Effects Model for Association of Longitudinal Cognition With 4 Marital Status Groups eTable 5. Linear Mixed-Effects Model for Association of Longitudinal Cognition With 4 Marital Status Groups Interacting With PiB eTable 6. Linear Mixed-Effects Models for the Association of Longitudinal Geriatric Depression Scores With 3 Marital Status Groups eTable 7. Linear Mixed-Effects Models for the Association of PACC Component Test Performance With 3 Marital Status Groups eFigure. Association of Longitudinal Cognition With 4 Marital Status Groups eReferences. [file jamanetwopen-3-e200121-s001.pdf]

Supplementary Online Content

Biddle KD, Jacobs HIL, d'Oleire Uquillas F, et al. Associations of widowhood and β-amyloid with cognitive decline in cognitively unimpaired older adults. *JAMA Netw Open*. 2020;3(2):e200121. doi:10.1001/jamanetworkopen.2020.0121

eAppendix. Supplemental Descriptions of Participants, Marital Status, and Emotional Support Score
eTable 1. Social Engagement, Physical Activity, and Close Relationship Data for the Sample
eTable 2. Baseline Medical, Psychiatric, and Health Behavior Data for the Sample
eTable 3. Linear Mixed-Effects Model for Association of Longitudinal Cognition With Dichotomous PiB-Marital Status Groups
eTable 4. Linear Mixed-Effects Model for Association of Longitudinal Cognition With 4 Marital Status Groups
eTable 5. Linear Mixed-Effects Model for Association of Longitudinal Cognition With 4 Marital Status Groups Interacting With PiB
eTable 6. Linear Mixed-Effects Models for the Association of Longitudinal Geriatric Depression Scores With 3 Marital Status Groups
eTable 7. Linear Mixed-Effects Models for the Association of PACC Component Test Performance With 3 Marital Status Groups
eFigure. Association of Longitudinal Cognition With 4 Marital Status Groups
eReferences.

This supplementary material has been provided by the authors to give readers additional information about their work.

© 2020 Biddle KD et al. *JAMA Network Open.*

eAppendix. Supplemental Descriptions of Participants, Marital Status, and Emotional Support Score

Methods:

Participants
All participants were cognitively unimpaired at enrollment based on a Clinical Dementia Rating global score of 0 and performance within education-adjusted norms on the Wechsler Logical Memory delayed recall subtest and the Mini-Mental State Examination (MMSE). At baseline, medical, neurological and psychiatric history were surveyed by participant self-report, corroborated by the study physician and, if necessary, by medical record review. Participants with past or present major psychiatric disorders were excluded, although a history of remitted mild depression was allowed.

Participants who had completed four annual study visits (three annual follow up visits; n=257) were included in the sample. Twenty-four participants (13 married, 4 widowed, 7 unmarried) were not included in the sample because they dropped out before completing their fourth annual visit (4 with 0 follow-up, 11 with 1 year follow up, 11 with 2 year follow up). Among the 281 participants marital group was not associated with attrition or non-attrition (i.e., non-inclusion or inclusion in the sample) by Fisher's exact test (p=0.9).

Marital Status
All participants reported their current marital status at the baseline study visit by selecting one of the following marital categories: "Single," "Married," "Divorced," "Separated," "Widowed," "Never Married," "Unknown," or "Other"**.** No participants chose "Unknown" or "Other". One participant selected two categories ("Divorced" and "Widowed") and was classified as "Widowed". Based on available data for 27 widowed participants, the mean duration of widowhood at baseline was 9.9 years (median 14.4, range 2.4-55). Twenty-one participants from the sample changed marital status over 3 years. This included 8 married participants who became widowed and 4 widowed participants who became married.

Cognitive Function
Out of the full baseline sample of 257 participants, 22 participants lacked valid data for the Free and Cued Selective Reminding Test at baseline due to a test administration error. For these 22 participants, the Preclinical Alzheimer Cognitive Composite z-score at baseline was computed as the mean z-score from the other three test components (the Mini-Mental State Examination, the Logical Memory test and the Digit Symbol Coding).

Emotional Support
Level of emotional support from children, relatives and friends was assessed by three questionnaire items derived from the MacArthur Studies of Aging[1], asking participants to specify numbers of close relationships as follows: "If you have children, how many of your *children* do you feel close to? In general, apart from children, how many *close relatives* do you have who you feel at ease with, can talk to about private matters and can call on for help? In general, how many *close friends* do you have who you feel at ease with, can talk to about private matters and can call on for help?' Emotional support data were acquired at the fourth annual assessment. As these data were not collected at earlier assessments, for the purpose of this study these responses were used as proxy baseline data.

© 2020 Biddle KD et al. *JAMA Network Open.*

eTable 1. Social Engagement, Physical Activity, and Close Relationship Data for the Sample

	**Overall (N=257)**		**Married (N=145)**		**Unmarried (N=77)**		**Widowed (N=35)**		***p*-value**
	Value	Range	Value	Range	Value	Range	Value	Range	Tukey or chi square test
Social Engagement	8.4 (4.5)	0-23	7.5 (4.0)	0-20	9.7 (5.2)	0-23	9.5 (3.9)	0-17	**UM-M: *p*=0.002** **W-M: *p*=0.04** W- UM: *p*=0.98
Physical Activity Score	17.7 (12.8)	0-70	19.2 (13.8)	0-70	15.3 (10.4)	0-45	16.8 (12.5)	0-60	UM-M: *p*=0.08 W-M: *p*=0.59 W- UM: *p*=0.83
Number of Close Relationships	7.9 (4.8)	0-28	7.9 (4.7)	0-28	7.5 (5.3)	0-28	8.5 (4.6)	2-24	UM -M: *p*=0.87 W-M: *p*=0.80 W- UM: *p*=62

Abbreviations: M, Married; UM, Unmarried; W, Widowed. Data is reported as mean (standard deviation) values or as number and percentage.

© 2020 Biddle KD et al. *JAMA Network Open.*

eTable 2. Baseline Medical, Psychiatric, and Health Behavior Data for the Sample

Condition or Health Related Factor	Married (n=145)	Unmarried (n=77)	Widowed (n=35)	Group differences
Hypertension	65 (45%)	38 (49%)	15 (43%)	*p*=0.77
Diabetes Mellitus	9 (6%)	10 (13%)	4 (11%)	*p*=0.21
Myocardial infarction or Cardiac Bypass Surgery	6 (4%)	3 (4%)	2 (6%)	*p*=0.83
Cerebrovascular Accident or Transient Ischemic Attack	5 (3.5%)	2 (2.5%)	0	*p*=0.87
Hearing loss (self-reported loss with or without hearing aid)[a]	76 (52%)	40 (52%)	17 (49%)	*p*=0.81
Alcohol Abuse	7 (5%)	1 (1%)	1 (3%)	*p*=0.40
Substance Abuse	1 (0.5%)	0	0	*p*=1
Depression	20 (14%)	13 (17%)	6 (17%)	*p*=0.78
Current Alcohol Use				UM -M: *p*=0.66<br>W-M: *p*=0.73<br>W- UM: *p*=0.99
None	28 (19%)	22 (29%)	9 (26%)	
<1 drink/day	79 (54%)	35 (45%)	18 (51%)	
1-2 drinks/day	27 (19%)	19 (25%)	8 (23%)	
3-4 drinks/day	6 (4%)	1 (1%)	0	
Current Smoking	4 (3%)	2 (3%)	1 (3%)	*p*=1
Current SSRI/SNRI medication use	21 (14%)	5 (6.5%)	1 (3%)	*p*=0.07

Abbreviations: M, married; SSRI/SNRI, Selective Serotonin Reuptake Inhibitor/Serotonin Norepinephrine Reuptake Inhibitor; UM, unmarried; W, widowed. Data for 257 participants are shown. Except as otherwise noted, data represent number and percent positive for any lifetime history of the health condition. Group differences were tested by the Chi Square, Fisher's exact test and Tukey HSD test.

© 2020 Biddle KD et al. *JAMA Network Open.*

eTable 3. Linear Mixed-Effects Model for Association of Longitudinal Cognition With Dichotomous PiB-Marital Status Groups

**Model: Longitudinal PACC Scores**	**β Estimate (95% CI)**	***t* value**	***p* value**	**Effect size (Cohen's d)**
Unmarried PiB- × Time (ref=Married PiB-)	-0.05 (-0.11 to 0.01)	-1.55	0.12	0.2
Widowed PiB- × Time (ref=Married PiB-)	-0.09 (-0.17 to -0.009)	-2.15	**0.03**	0.27
Married PiB+ × Time (ref=Married PiB-)	-0.12 (-0.18 to -0.06)	-3.7	**<0.001**	0.27
Unmarried PiB+ × Time (ref=Married PiB-)	-0.10 (-0.18 to -0.02)	-2.47	**0.01**	0.18
Widowed PiB+ × Time (ref=Married PiB-)	-0.33 (-0.46 to -0.19)	-4.56	**<0.001**	0.58
Baseline Age × Time	-0.004 (-0.008 to -0.0001)	-2.01	**0.04**	0.26

Abbreviations: GDS, Geriatric Depression Scale; PACC, Preclinical Alzheimer's Composite; PiB, Pittsburgh Compound-B. Model included 254 participants and 1015 observations. Results for predictors of interest and covariates associated with longitudinal cognition (*p*<0.1) are shown.

© 2020 Biddle KD et al. *JAMA Network Open.*

eTable 4. Linear Mixed-Effects Model for Association of Longitudinal Cognition With 4 Marital Status Groups

**Model: Longitudinal PACC Scores**	**β Estimate (95% CI)**	***t* value**	***p* value**	**Effect size (Cohen's d)**
Widowed × Time (ref=Married)	-0.11 (-0.19 to -0.04)	-3.07	**0.002**	0.22
Divorced/Separated × Time (ref=Married)	-0.02 (-0.08 to 0.04)	-0.61	0.55	0.04
Single/Never Married × Time (ref=Married)	-0.05 (-0.12 to 0.02)	-1.48	0.14	0.11
Baseline PiB Binding × Time	-0.14 (-0.20 to -0.08)	-4.54	**<0.001**	0.33
Baseline Age × Time	-0.004 (-0.008 to 0.0001)	-1.88	0.06	0.14

Abbreviations: GDS, Geriatric Depression Scale; PACC, Preclinical Alzheimer's Composite; PiB, Pittsburgh Compound-B. Model included 254 subjects and 1015 observations. Results for predictors of interest and covariates associated with longitudinal cognition ($p<0.1$) are shown.

© 2020 Biddle KD et al. *JAMA Network Open.*

eTable 5. Linear Mixed-Effects Model for Association of Longitudinal Cognition With 4 Marital Status Groups Interacting With PiB

**Model: Longitudinal PACC Scores**	**β Estimate (95% CI)**	***t* value**	***p* value**	**Effect size (Cohen's d)**
Widowed × PiB Binding × Time (ref=Married)	-0.22 (-0.41 to -0.03)	-2.25	**0.02**	0.29
Divorced/Separated × PiB Binding × Time (ref=Married)	-0.05 (-0.20 to 0.11)	-0.59	0.56	0.08
Single/Never Married × PiB Binding × Time (ref=Married)	0.06 (-0.13 to 0.25)	0.63	0.53	0.08
Baseline Age × Time	-0.004 (-0.008 to 0.0002)	-1.84	0.07	0.24

Abbreviations: GDS, Geriatric Depression Scale; PACC, Preclinical Alzheimer's Composite; PiB, Pittsburgh Compound-B. Model included 254 subjects and 1015 observations. Results for predictors of interest and covariates associated with longitudinal cognition (p<0.1) are shown.

© 2020 Biddle KD et al. *JAMA Network Open.*

eTable 6. Linear Mixed-Effects Models for the Association of Longitudinal Geriatric Depression Scores With 3 Marital Status Groups

Model: Longitudinal GDS Scores	β Estimate (95% CI)	*t* value	*p* value	Effect size (Cohen's d)
Widowed × Time (ref=Married)	-0.14 (-0.54 to 0.26)	-0.67	0.50	0.05
Unmarried × Time (ref=Married)	-0.12 (-0.40 to 0.16)	-0.83	0.41	0.06
Baseline PiB Binding × Time	0.31 (-0.01 to 0.64)	1.86	0.06	0.14

Abbreviations: GDS, Geriatric Depression Scale; PACC, Preclinical Alzheimer's Composite; PiB, Pittsburgh Compound-B. Model included 254 subjects and 1012 observations. Model adjusted for age, sex, socioeconomic status, PiB and their interactions with time. Results for predictors of interest and covariates associated with longitudinal cognition (p<0.1) are shown.

© 2020 Biddle KD et al. *JAMA Network Open.*

eTable 7. Linear Mixed-Effects Models for the Association of PACC Component Test Performance With 3 Marital Status Groups

**Dependent Variable: Logical Memory Test**	**β Estimate (95% CI)**	***t* value**	***p* value**	**Effect size (Cohen's d)**
Widowed × Time (ref=Married)	-0.53 (-0.96 to -0.09)	-2.37	0.02	0.14
Unmarried × Time (ref= Married)	-0.31 (-0.61 to 0.002)	-1.93	0.05	0.17
**Dependent Variable: FCSRT**	**β Estimate (95% CI)**	***t* value**	***p* value**	**Effect size (Cohen's d)**
Widowed × Time (ref= Married)	-0.27 (-0.48 to -0.07)	-2.56	0.01	0.19
Unmarried × Time (ref= Married)	-0.01 (-0.16 to 0.13)	-0.16	0.88	0.01
**Dependent Variable: MMSE**	**β Estimate (95% CI)**	***t* value**	***p* value**	**Effect size (Cohen's d)**
Widowed × Time (ref= Married)	-0.09 (-0.24 to 0.07)	-1.07	0.28	0.08
Unmarried × Time (ref= Married)	-0.03 (-0.15 to 0.08)	-0.61	0.54	0.04
**Dependent Variable: Digit Symbol Test**	**β Estimate (95% CI)**	***t* value**	***p* value**	**Effect size (Cohen's d)**
Widowed × Time (ref= Married)	-0.61 (-1.55 to 0.32)	-1.27	0.20	0.09
Unmarried × Time (ref= Married)	-0.22 (-0.88 to 0.45)	-0.63	0.53	0.05

Abbreviations: FCSRT, Free and Cued Selective Reminding Test; GDS, Geriatric Depression Scale; MMSE, Mini Mental State Examination. Models included 254 participants and 1014 observations, 254 participants and 993 observations, 254 participants and 1015 observations, and 254 participants and 1014 observations, respectively. All models adjusted for age, sex, Hollingshead and Geriatric Depression Scale scores, Depression history and Pittsburgh Compound B binding and each of their interactions with time. Marital status predictors of interest are shown.

© 2020 Biddle KD et al. *JAMA Network Open.*

eFigure. Association of Longitudinal Cognition With 4 Marital Status Groups

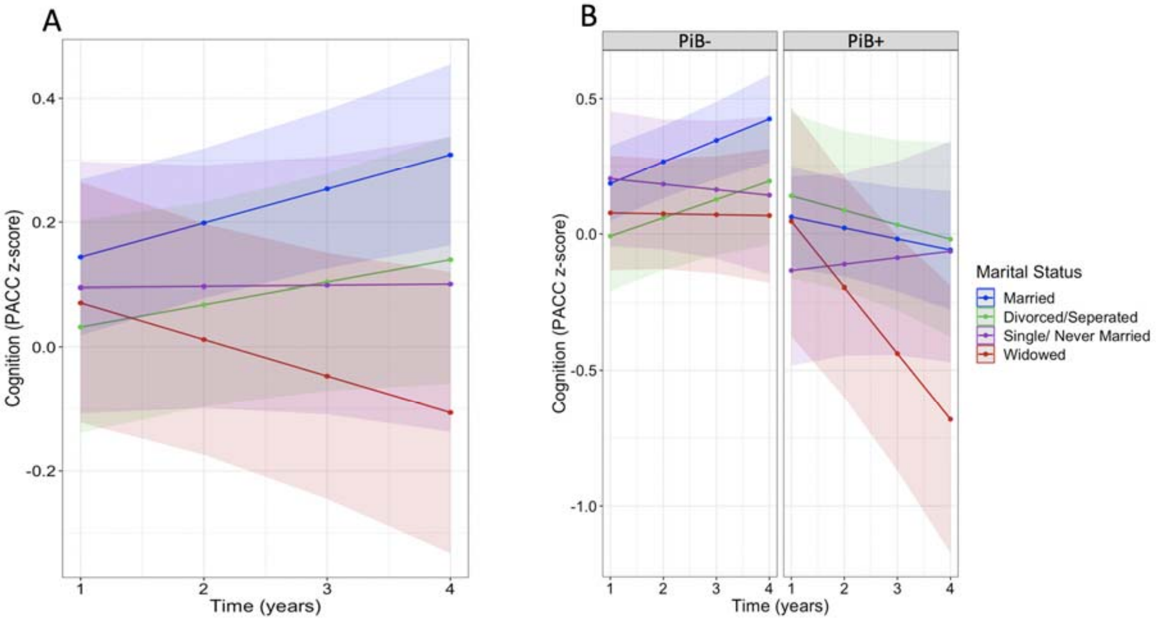

eReferences.

eFigure 1 A) PACC scores declined in widowed participants (red) over 3 years compared to married (blue) participants, controlling for age, sex, socioeconomic status, depression history, depressive symptoms, neocortical amyloid-β, and their effects by time. Longitudinal PACC scores for Divorced/Separated participants (green) and Single/Never Married (purple) participants did not differ from Married (blue) participants. **B)** To illustrate the interactive effects of widowhood and PiB on change in cognition, predicted trajectories for the four marital status groups are shown separately for low amyloid-β (PiB-) and high amyloid-β (PiB+) groups, controlling for the same covariates as in Panel A. PiB+ is based on the standard cutoff > 1.32. 95% confidence intervals are shown.
.

© 2020 Biddle KD et al. *JAMA Network Open.*

eReferences.

Seeman TE, Lusignolo TM, Albert M, Berkman L. Social relationships, social support, and patterns of cognitive aging in healthy, high-functioning older adults: MacArthur studies of successful aging. *Health Psychol.* 2001;20(4):243-255.
